# Trajectories of poverty and economic hardship among American families supporting a child with a neurodisability

**DOI:** 10.1111/jir.12666

**Published:** 2019-07-12

**Authors:** D. W. Rothwell, G. Gariépy, F. J. Elgar, L. M. Lach

**Affiliations:** ^1^ Human Development &Family Sciences, College of Public Health and Human Sciences Oregon State University Corvallis OR USA; ^2^ Department of Social and Preventive Medicine University of Montreal Montreal QC Canada; ^3^ Institute for Health and Social Policy McGill University Montreal QC Canada; ^4^ School of Social Work McGill University Montreal QC Canada

**Keywords:** economic hardship, longitudinal study, neurodisability, poverty, trajectories

## Abstract

**Background:**

Caring for a child with a neurodisability (ND) impacts the financial decisions, relationships and well‐being of family members, but evidence on the economic trajectories of families throughout the life course is missing.

**Methods:**

Using data from the Panel Study of Income Dynamics, we tracked the families of 3317 children starting 5 years before childbirth until the child reached 20 years of age. We used regression and latent growth curve modelling to estimate trajectories of poverty and economic hardship over time.

**Results:**

Families with a child with an ND had higher rates of poverty and economic hardship prior to childbirth and persistently over time. Analysis uncovered five latent trajectories for each indicator. After controlling for family and caregiver characteristics that preceded the birth of the child, raising a child with an ND was not associated with a unique trajectory of poverty. Families raising a child with an ND were however more likely to experience persistent economic hardship.

**Conclusions:**

The study establishes descriptive evidence for how having a child with an ND relates to changes in family economic conditions. The social and economic conditions that precede the child's birth seem to be driving the economic inequalities observed later throughout the life course.

## Background

The past 50 years have witnessed a major increase in childhood disabilities, driven by a rise in the proportion of neurodevelopmental and behavioural disorders, while the proportion of medical and physical disabilities have declined (Halfon *et al*. 2012). Presently, one in 10 families in North America care for a child with a neurodisability (ND), defined as ‘congenital or acquired long‐term conditions that are attributed to impairment of the brain and/or neuromuscular system’ (Morris *et al*. 2013, p. 1103), such as a learning disability, intellectual disability (ID) or autism spectrum disorder (Lach *et al*. 2009; Arim *et al*. 2012; Zablotsky *et al*. 2015). Recent estimates from the National Health Interview Survey reveal that families with a child with an ND are more likely to be living in poverty than other families (Halfon *et al*. 2012).

For several reasons, the economic, social and health burdens of NDs may deepen and compound the impact of poverty. Yet understanding poverty as a dichotomous and static oversimplifies the condition. Seminal work on poverty dynamics in the general population found that many families have short spells of poverty, and a much smaller proportion experience long duration of poverty (e.g. Bane & Ellwood 1986). Specifically, the addition of a child to a household increases the risk of poverty because children increase the resource needs without adding resources. Little is known about the poverty dynamics of families raising children with an ND.

Two major frameworks are useful for explaining health and economic inequalities across the life course. The first is a *health selection path*. In this view, differences in health status directly affect socio‐economic position (Kröger *et al*. 2015). The idea is that health enables greater social and economic achievements over time, while the opposite trajectory holds for ill health. In the context of an ND, the health selection framework suggests that the presence of child with an ND will lead to less favourable socio‐economic outcomes for the family. Studies have shown that caring for a child with ND is expensive in terms of direct expenses of managing the condition and indirect costs in terms of the time needed to care for the child (Anderson *et al*. 2007; Stabile & Allin 2012; Lavelle *et al*. 2014; Genereaux *et al*. 2015; Saunders *et al*. 2015). Maternal labour activity (i.e. hours worked) may be particularly affected when a child has a disability, chiefly during early childhood (Porterfield 2002; Wehby & Ohsfeldt 2007; Burton & Phipps 2009; Lu & Zuo 2010; Stabile & Allin 2012). Saunders *et al*. (2015) found that about two‐thirds of mothers of children with an ND reduced their work hours or stopped working altogether to care for their child. Cidav *et al*. (2012) found that mothers of children with autism spectrum disorder worked 7 h less per week and earned 56% less on average than other mothers. Family structure is another pathway through which disability is socially stratified. Single parent families are at greater risk for poverty by way of having one less income earner and less help with household and child‐rearing tasks. Mothers of children with ND are less likely to marry and those who do are more likely to divorce than mothers of children without an ND (Mauldon 1992; Joesch & Smith 1997; Kvist *et al*. 2013). Other studies demonstrated that children with early cognitive delay were less likely than other children to live with both biological parents and more likely to experience changes in family composition in the first 5 years (Hatton *et al*. 2010), although the effects of changes in family composition were similar for both groups. Although, more recent studies showed that children with autism spectrum disorder, compared with children without, were at no greater risk for children to live in households without two biological or adoptive parents (Freedman *et al*. 2012).

The second framework is *social causation*, which suggests that it is the poorer socio‐economic conditions that increase the risk of health problems. Limited access to resources, social support and knowledge stratifies population health (Power *et al*. 2002; Palloni *et al*. 2009). When applied to families who have a child with an ND, the social causation model would predict that the economic consequences typically associated with the ND would not be explained by the child's health status *per se* but by the pre‐existing socio‐economic position. Delobel‐Ayoub *et al*. (2015) found that prevalence of isolated and severe ID was geographically concentrated. In other words, families who are worse off may be more likely to have a child with an ND and continue to fare worse thereafter than those from more privileged socio‐economic conditions.

Although the evidence of association between childhood disability and poverty is irrefutable (Stabile & Allin 2012), the direction of influence remains unclear because of common social and economic factors that contribute to – and result from – child NDs. While previous research has examined poverty trajectories and health across the life course (McDonough *et al*. 2005), few have examined the poverty risk for families raising children with an ND. The few longitudinal studies point to mixed results that depend on the economic resources measured, selected poverty thresholds and study duration. For example, in a study over 4 years (2001–2005), Shahtahmasebi *et al*. (2011) found a positive association between families supporting a child with a disability and poverty duration, yet this disappeared when contemporaneous family characteristics such as family structure, age and health status of caregiver were controlled. Using the same survey data set, Shahtahmasebi *et al*. (2010) showed that child disability was positively related to a material hardship scale but this relationship was reversed for income poverty. Yet, in another study over a 1‐year period, families supporting a child with a disability were significantly more likely to experience poverty and hardship (Emerson *et al*. 2010). The extent to which caring for a child with an ND places families at greater economic hardship and risk of poverty over a longer run of the life course remains unclear.

The objectives of this study were therefore (1) to examine the risk of poverty for families raising a child with an ND over time, (2) to estimate the trajectories of poverty that families experience over time and (3) to investigate whether having a child with an ND was associated with trajectories that experience greater poverty risk over time. In addition to poverty, we also measured a less conservative indicator of economic hardship. We exploited data from a long‐run intergenerational panel study that measured socio‐economic conditions of families before having a child with an ND up to 25 years later. Although we do not expect either framework to fully explain the patterns over time, given magnitude of gaps observed in previous work, we hypothesised that families of children with an ND would be more likely to be poor and experience economic hardship at any given time and that families with a child with ND would follow less favourable poverty and hardship trajectories. Our analysis of pre‐birth socio‐economic conditions is essential to sorting the relative importance of the health selection versus social causation frameworks in explaining socio‐economic disparities of families raising a child with ND.

## Methods

### Study population

The Panel Study of Income Dynamics (PSID) is a nationally representative longitudinal household survey of over 9000 American families followed annually from 1968 to 1997 and biennially from 1997 to the present (Institute for Social Research 2015). The PSID is a genealogical sample in that the original individuals and households from the 1968 sample have passed down the PSID ‘gene’. Individuals without the PSID gene are included in the study so long as they live with a PSID member. This means that most PSID participants have a relationship – biological or other – to the original 1968 sample (for more details on PSID, see McGonagle *et al*. 2012). In 1997, the PSID initiated the Child Development Supplement (CDS) to collect information on children of the cohort. Three waves of CDS data are available. The CDS‐I (1997–1998) completed interviews with 2394 families, providing information on 3563 children aged 0–12 years (88% response rate). PSID families with children aged 0–12 years were randomly sampled, with up to two children per family. In‐person interviews were conducted with the primary caregivers, and assessments were conducted with children. The CDS‐II (2002–2003) re‐interviewed 2901 children (84% of those eligible), and the CDS‐III (2007–2008) re‐interviewed 1506 children (90% of those eligible). We first built a sample of CDS participants that were between 16 and 28 years old. We excluded children with missing information on year of birth (*n* = 246) for a final sample size of 3317 children. We then linked those children to their family unit via the ID number of the parents and child across years 1968 to 2013. To simplify, we delimited the sample to 5 years before the birth to age 20 years for a total of 25 years of family information per child. The final analytical sample covered 82 925 family‐years (i.e. the number of family observations across all years).

### Measures

#### Neurodisability

In the US context, a learning disability is typically considered a distinct condition that is separate from others such as cognitive impairment and a low IQ (for a review of the definitional issue, see Moll *et al*. 2014). However, to be consistent with previous research on family outcomes among families with a child with an ND (Lach *et al*. 2009; Garner *et al*. 2013; Morris *et al*. 2013; Arim *et al*. 2017), we applied a broader classification that combined learning and other NDs. Specifically, in the CDS, parents were asked about whether their child had been diagnosed with a list of health conditions. A child was coded as having an ND if parents reported any of the following six conditions: epilepsy, speech impairment, retardation, developmental delay, autism and learning disability.

#### Family poverty and economic hardship

The PSID collected detailed information on total family income at every survey wave. The PSID defines families as economically independent households whose members share household finances and are related by birth/adoption, marriage or cohabitation. Family income is the sum of all taxable income, transfer income and social security income of all family unit members. We analysed two economic indicators. The first was absolute family poverty using year‐specific national poverty threshold (United States Census Bureau 2015). This official poverty measure considers most sources of cash income as resources and uses a family‐size adjusted need threshold. We categorised families as poor if the family income fell below the official poverty threshold. Because the official poverty measure might not accurately reflect need in contemporary society and prior research showing scale of poverty and economic hardship leads to different results (Shahtahmasebi *et al*. 2010), we constructed a second indicator of economic hardship based on the income‐to‐needs ratio. This ratio is defined as family income relative to the poverty threshold, where 1.0 represents a family income at the poverty line. We set the family economic hardship at 150% or below the income‐to‐needs ratio based on previous poverty research (Shaefer *et al*. 2015). Broader indicators of poverty such as our measure of economic hardship are often used to determine eligibility for social assistance.

#### Covariates

Several baseline sociodemographic factors could confound the association between having a child with an ND and the trajectories of family income. To account for this, we included the following covariates for the primary caregiver and household measured in the year prior to childbirth: age, race/ethnicity, years of education, marital status (married/living with partner vs. not), employment status (currently working vs. not), self‐rated health (good/very good/excellent vs. fair/poor) and number of children. We did not control for the gender of the primary caregiver because a large majority (97.1%) were women. We also measured child gender and year of birth of the child.

### Statistical analysis

We defined time zero as the birth year of the child. In a first step, we conducted pooled logistic regressions to model the probability of family poverty and economic hardship over time (i.e. by the age of the child) by ND status. We included ND status, time (age) and an interaction between time and ND status to allow the association between ND and poverty to vary over time. We then plotted the predicted probabilities of poverty and economic hardship by time and ND status. In complementary analyses (Fig. S1), we also applied latent growth models using a second‐order polynomial to graph changes in poverty and economic hardship by ND status over time. A comparison of the pooled logistic results and the unconditional latent growth models appears in Tables S1 and S2.

In a second step, following previous work on income dynamics (Wolf *et al*. 2014), we identified distinct poverty and hardship trajectories from latent class growth modelling (LCGM) (Andruff *et al*. 2009) using the *traj* command in stata 14 (Jones *et al*. 2001). LCGM is a semi‐parametric technique used to uncover distinct groups that follow a similar pattern of change over time. LCGM is sometimes referred to as group‐based trajectory analysis and is noted for its person‐based (as opposed to variable‐based) approach for the exploration of development outcomes across time (Nagin 1999). The procedure assumes that missing data on the dependent variable are missing at random and therefore includes subjects with some missing data on the dependent variable in analysis. The LCGM incorporated sampling weights at the household level from the CDS. We started by fitting a one‐class solution, equivalent to the hypothesis that all families followed the trajectory of economic hardship or poverty and added more classes until the model reached a good fit with the data. We selected the number of trajectories based on the Bayesian information criterion, interpretability of the model and meaningfulness of each class (Nagin 1999). We used second‐order polynomial terms to allow for non‐linearity and selected the form of the trajectories in the final model based on the significance of the polynomial components of each class. Using a two‐step class enumeration approach, we first classified participants in the trajectory group for which they had the highest probability of belonging. We then examined whether having a child with ND and family sociodemographic factors predicted the type of poverty trajectory that family would likely experience for the next 20 years by running multinomial logistic regressions. The two‐step approach was chosen over other methods because the first step allows us to model growth curves for all families and subsequently examine how ND status was associated with the various trajectories. We repeated the same steps to identify trajectories for economic hardship. Statistical analyses were conducted in stata (version 14.1, Stata Corp., College Station, TX).

## Results

We identified 656 children with an ND in the sample (weighted prevalence 20.3%). Table 1 presents socio‐economic characteristics of families with and without a child with an ND at baseline (birth year of child). Primary caregivers of children with an ND had similar sociodemographic profiles to caregivers of children without an ND but were less likely to be married, were less likely to work and were more likely to report fair or poor health. As expected, more families of children with an ND were economically disadvantaged at baseline (i.e. year of childbirth) with lower median household income and a smaller income‐to‐needs ratio compared with non‐ND families. ND families also had a greater prevalence of poverty at 18.3% [95% confidence interval (CI) 13.9, 22.6%] compared with 11.5% (95% CI 9.8, 13.2%) in those without a child with an ND, a significant difference of 6.8 percentage points (95% CI 2.0, 11.4). Further, ND families experience greater economic hardship, with 30.4% (95% CI 25.2, 35.6) of ND families below the 150% threshold compared with 19.7% (95% CI 17.6, 21.9) of non‐ND families.

**Table 1:** Characteristics of study participants at baseline (birth year of the child) by ND status (n = 3317)

	Children with an ND (n = 656)		Children without an ND (n = 2661)		
	Weighted % (n)	Weighted mean (SD)	Weighted % (n)	Weighted mean (SD)	
Primary caregiver					
Age (years)		27.78 (7.25)		28.15 (6.17)	
Gender					
Female	97.5 (639)		97.0 (2585)		
Race/ethnicity					
White	71.6 (338)		69.3 (1268)		
Black	15.8 (253)		16.1 (1135)		
Other	12.6 (58)		14.6 (249)		
Marital status					
Married/partnered	78.5 (387)		82.5 (1655)		
Not married/partnered	21.5 (157)		17.5 (614)		
Years of education		13.37 (2.46)		13.88 (2.53)	
Working status					
Working	42.3 (220)		50.7 (1036)		
Not working	57.7 (298)		49.3 (1069)		
Self‐rated health					
Good/very good/excellent	88.8 (474)		93.9 (2025)		
Fair/poor	11.2 (68)		6.1 (236)		
Household					
Number of children		1.82 (1.20)		1.62 (1.07)	
Income (median, in 2013 USD)		49 836 (47 646)		61 207 (57 509)
Income‐to‐needs ratio		3.06 (2.51)		3.66 (2.91)	
Living in poverty	18.3 (137)		11.5 (409)		
Living in economic hardship	30.4 (208)		19.7 (642)		

Weighted using household survey weights.

ND, neurodisability; SD, standard deviation.

**Figure 1:**
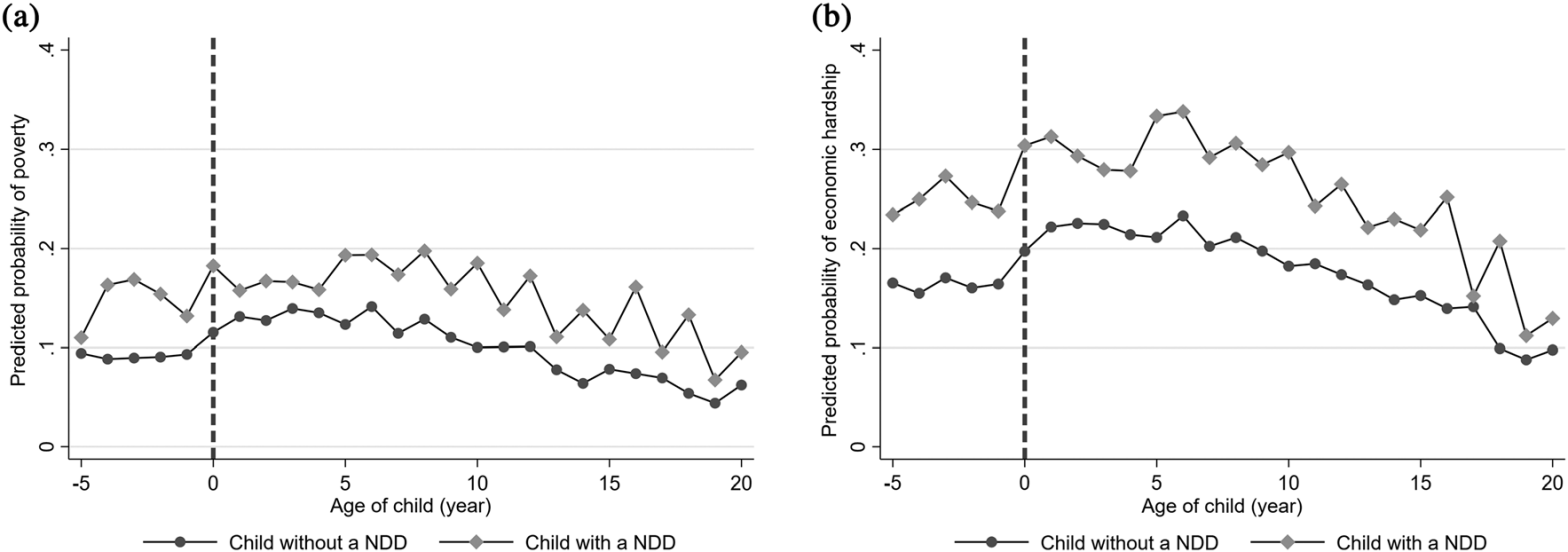
(a) Predicted probability of family living below the poverty threshold; (b) predicted probability of family living at or below 150% income‐to‐needs ratio. Models were weighted using household survey weights. Bold dash indicates year of birth (age 0).

Using a pooled logistic regression model, Fig. 1 illustrates the risk of poverty and economic hardship for ND families and non‐ND families before childbirth and across the course of the child's development. At 5 years before birth, we found little difference in the poverty risk across groups (difference of 1.6%, 95% CI −2.2, 5.3). Closer to childbirth economic circumstances begin to show differences. Families who would have a child with an ND experienced on average a 5.4% (95% CI 3.5, 7.3) higher probability of poverty across the 5‐year period before birth compared with other families. During the 20 years postnatal, families of children with an ND continued to have an average 5.1% (95% CI 3.9, 6.2) higher probability of poverty year over year (Fig. 1a). Consistent with previous research on the risk of poverty across the life cycle (Rank & Hirschl 2001), the risk of poverty was higher in the younger child years and fell around the time the child reached 10 years old. (The poverty risk across child age is consistent, comparable across the pooled logistic and latent growth curve models; see Fig. 1a and Tables S1 and S2.)

Differences between groups over time were also observed for economic hardship (Fig. 1b). Because the economic hardship indicator used a more relaxed cut‐off, the prevalence of hardship was greater than that of poverty. During the 5 years prior to childbirth, ND families had on average an 8.5% (95% CI 6.1, 10.9%) higher probability of experiencing hardship compared with non‐ND families. Figure 1b shows how the risk of economic hardship fell with the age of the child while the differences between groups remained mostly constant across time. From birth to age 20 years, the average predicted probability of economic hardship was 8.2% (95% CI 6.8, 9.6) higher for ND families compared with non‐ND families. (The economic hardship risk across child age is consistent, comparable across the pooled logistic and latent growth curve models; see Fig. 1b and Tables S1 and S2).

**Figure 2:**
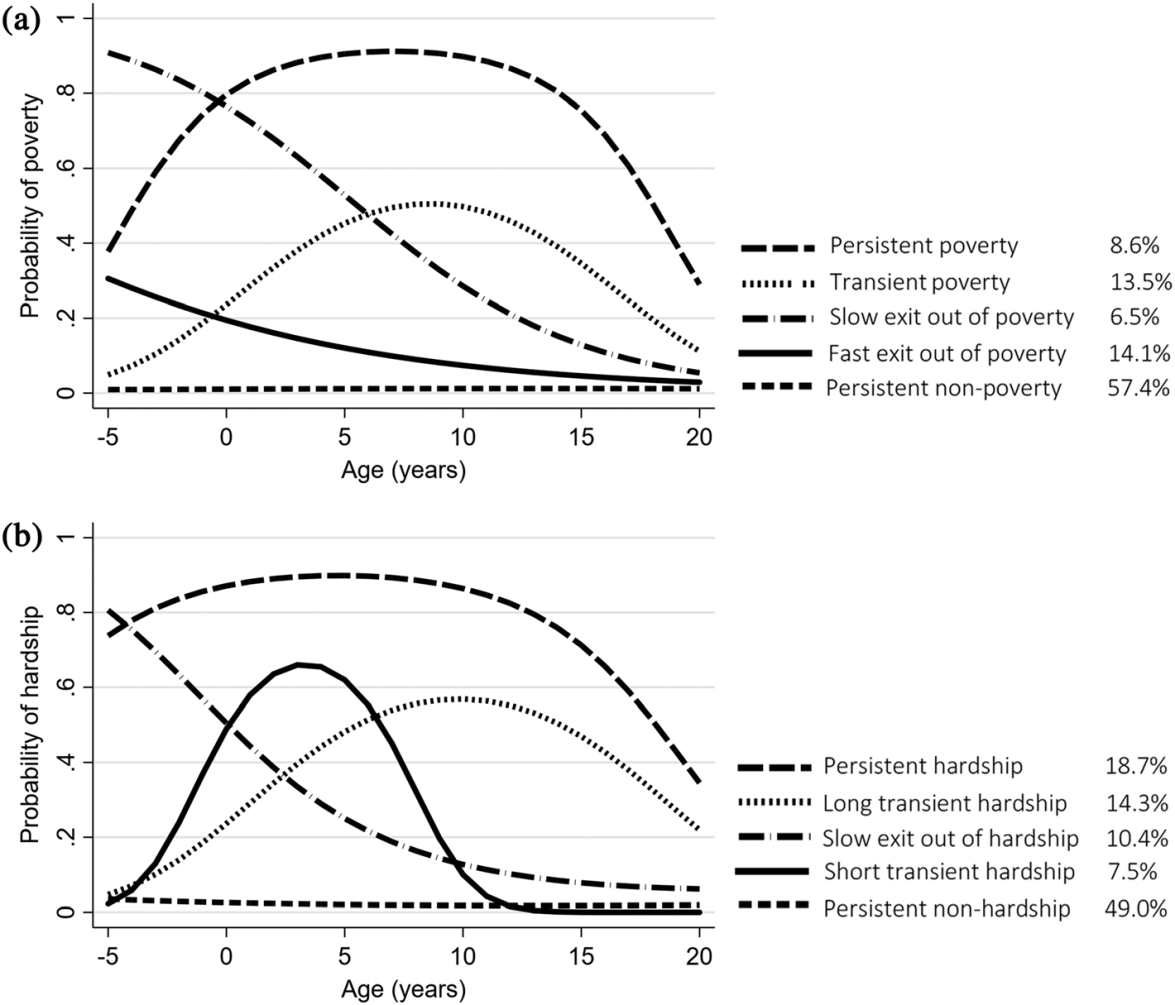
(a) Trajectories of probability of family living below the national poverty threshold; (b) trajectories of probability of family living below 150% of the income‐to‐needs ratio.

Turning to trajectories of family poverty, the LCGMs suggested five distinct trajectory patterns for the period ranging from 5 years before to 20 years after childbirth. Of the models run, we chose a five‐class model, based on model fit, interpretability and meaningfulness of the classes (Fig. 2a; see Tables 3S and S4 for detailed model fit statistics and comparisons across other models). The first and largest trajectory was *persistent non‐poverty* (57.4% of the sample; average probability of class membership: 0.93, 99% CI 0.92, 0.93). Membership in this group was characterised by a constantly low likelihood of experiencing poverty. The second largest group was *fast exit out of poverty* (14.1% of the sample; average probability of class membership: 0.79, 99% CI 0.76, 0.81), which included families with moderate poverty risk in the early years that declined sharply over time. The *slow exit out of poverty* group (6.5%; average probability of class membership: 0.86, 99% CI 0.83, 0.89) had the highest probability of poverty before the childbirth, but this risk declined steadily. By the time the child reached the age of 6 years, this group had moderate risk of poverty. The remaining two groups saw increases in poverty risk over time until about age 10 years. The *transient poverty* group (13.5%; average probability of class membership: 0.83, 99% CI 0.81, 0.85) started the period with very low risk of poverty that increased steadily. Between age 7 and 20 years, this group had the second highest risk of poverty. Last was the *persistent poverty* group (8.6%; average probability of class membership: 0.93, 99% CI 0.91, 0.95). This group had the highest probability of being poor from the time of childbirth onwards.

Similar trajectory patterns were observed for economic hardship (Fig. 2b, model fit statistics and details in Tables S5 and S6). As expected, compared with poverty trajectories, fewer families were in *persistent non‐hardship* (49.0% of the sample; average probability of class membership: 0.95, 99% CI 0.94, 0.96). The *slow exit out of hardship* (10.4%; average probability of class membership: 0.89, 99% CI 0.87, 0.91) group was roughly comparable with the same group on poverty, with high initial risk of hardship 5 years prior to childbirth that declined steadily over time. The *persistent hardship* (18.7%; average probability of class membership: 0.93, 99% CI 0.92, 0.94) group started with a high probability that continued until around the child reached adolescence after which the likelihood declined but remained high. With the more relaxed threshold cut‐off, this group was, as expected, proportionately larger than the persistently poor group in Fig. 2a (18.7% vs. 8.6%). The *long transient hardship* (14.3%; average probability of class membership: 0.86, 99% CI 0.84, 0.90) matched the shape of trajectory for the transient poverty group with very low initial probability that peaked at about the age of 10 years. From age 8 years onward, this group experienced the second highest risk of hardship. Finally, the *short transient hardship* group (7.5%; average probability of class membership: 0.81, 99% CI 0.78, 0.84) experienced a spike in hardship risk from age −5 to 4 years after which the risk declined steeply and virtually disappeared by adolescence.

Examination of the socio‐economic characteristics of the trajectory groups provided a deeper understanding of the trajectories (Tables S7 and S8). Race/ethnicity of the primary caregiver was closely related to poverty risk. For example, approximately 79% of the *persistent poverty* group was non‐white while 78% of the *persistent non‐poverty* group was white. As expected, higher educated caregivers had less poverty risk. Family structure played a significant role among the three groups that showed dynamic movement in and out of poverty. The *fast exit out of poverty* group had 61% of members married or partnered at baseline, compared with 25% of the *slow exit out of poverty* group. The *persistent poverty* and *low exit out of poverty* groups had the highest proportions of children with an ND (29% and 33%, respectively).

Next, we estimated multinomial models to examine baseline predictors of the trajectories. Table 2 shows results for poverty, and Table 3 results for economic hardship. In model 1, compared with other families, families with a child with an ND had a greater probability of experiencing all poverty trajectories relative to *persistent non‐poverty*. After adjustment for family sociodemographic factors in the year preceding childbirth, the child's ND status was no longer significantly associated with a specific pattern of poverty trajectory (Table 2).

**Table 2:** Association between child ND status and trajectory of family poverty using national poverty threshold

	Model 1 ND only	Model 2 Adjusted for child and family covariates	Model 3 Adjusted for child, family and caregiver covariates
Trajectory	Child has ND RRR (95% CI)	Child has ND RRR (95% CI)	Child has ND RRR (95% CI)
Persistent non‐poverty	Reference	Reference	Reference
Fast exit out of poverty	1.50 (1.06, 2.14)	1.48 (0.99, 2.2)	1.26 (0.80, 2.00)
Transient poverty	1.68 (1.19, 2.38)	1.70 (1.08, 2.70)	1.41 (0.84, 2.36)
Slow exit out of poverty	2.28 (1.33, 3.93)	1.91 (1.05, 3.48)	1.88 (0.90, 3.93)
Persistent poverty	1.91 (1.21, 3.02)	1.87 (1.05, 3.33)	1.47 (0.63, 3.45)

Estimates from multinomial logistic regression. All models weighted using household survey weights.

Model 1 is unadjusted.

Model 2 is adjusted for gender and birth year of the child and number of children in the household.

Model 3 is adjusted for gender and birth year of the child; number of children in the household; age, race, years of education, marital status, working status and self‐rated health of primary caregiver at year of childbirth.

CI, confidence interval; ND, neurodisability; RRR: relative risk ratio.

**Table 3:** Association between child ND status and trajectory of family economic hardship

	Model 1 ND only	Model 2 Adjusted for child and family covariates	Model 3 Adjusted for child, family and caregiver covariates
Trajectory	Child has ND RRR (95% CI)	Child has ND RRR (95% CI)	Child has ND RRR (95% CI)
Persistent non‐hardship	Reference	Reference	Reference
Short transient hardship	1.53 (1.01, 2.32)	1.34 (0.81, 2.22)	1.13 (0.64, 1.99)
Long transient hardship	1.67 (1.21, 2.31)	1.49 (0.99, 2.25)	1.28 (0.80, 2.06)
Slow exit out of hardship	1.85 (1.24, 2.77)	1.74 (1.12, 2.69)	1.54 (0.92, 2.59)
Persistent hardship	1.79 (1.29, 2.49)	2.19 (1.44, 3.34)	2.15 (1.19, 3.87)

Estimates from multinomial logistic regression. All models weighted using household survey weights.

Model 1 is unadjusted.

Model 2 is adjusted for gender and birth year of the child and number of children in the household.

Model 3 is adjusted for gender and birth year of the child; number of children in the household; age, race, years of education, marital status, working status and self‐rated health of primary caregiver at year of childbirth.

CI, confidence interval; ND, neurodisability; RRR: relative risk ratio.

A mostly similar pattern was observed for economic hardship. The composition of the persistent hardship trajectory revealed strong patterns of disadvantage (Table S8). Those in the *persistent hardship* group were younger, more likely to be minority, with low years of education, more children and worse self‐rated health. At baseline, members of this group were much more likely to be unmarried than other trajectory groups.

Results from the regression models (Table 3) indicate that the presence of a child with an ND in the family was associated with higher likelihood of all economic hardship trajectories compared with the *non‐hardship* trajectory. After adjustment for child and family factors, families raising a child with ND remained 74% more likely than non‐ND families to follow a *slow exit out of hardship* and 119% more likely to experience *persistent hardship*. Even after adjustment for primary caregiver characteristics before birth, ND families were still 115% more likely to experience *persistent hardship* prior to and throughout the child's development.

## Discussion

This study used 25 years of data from a large longitudinal panel survey to investigate economic trajectories in families with and without a child with NDs. The results underscore the challenging economic conditions faced by families raising a child with an ND. First, children who had ND were more likely than children without an ND to be born into poor families. At the time of the child's birth, the ND families had a poverty rate that was 6 percentage points higher than other families and a rate of economic hardship that was 10 percentage points higher. This finding indicates a significant socio‐economic disparity even before the birth of a child who is diagnosed with an ND and supports the findings by Shahtahmasebi *et al*. (2010) that child disability may be an intervening effect rather than functioning as a direct mechanism. Simply put, families that would eventually have a child diagnosed with an ND were more likely to be poor and in economic hardship, which is consistent with the national statistics from the US Census (Brault 2010).

Second, while the risks of poverty and hardship declined somewhat during the subsequent 20 years after birth, the socio‐economic difference between ND and non‐ND families remained largely intact. In other words, in two decades, the families of children with an ND were never able to close the economic gap. The peak risk for poverty and hardship appeared to hit families when the child was around 5 years of age, when caregiving responsibilities and expenses were perhaps most demanding. The persistent gap is consistent with work showing relatively constant gaps in socio‐economic status between families with a child with autism spectrum disorder between 2002 and 2010 (Durkin *et al*. 2017).

Third, our analysis of poverty trajectories revealed certain baseline characteristics of families in persistent poverty and hardship, who tended to be younger, with lower levels of human capital (levels of education), were more often single parents (i.e. not married or partnered) and were more likely to be in poor health.

Fourth, we show that results are somewhat sensitive to whether the outcome was poverty or economic hardship. No differences in trajectories were observed for poverty using the country's official poverty measure, but having a child with an ND put families at greater risk of persistent economic hardship (defined as 150% of the poverty line), even after adjusting for child, family and caregiver characteristics. Caring for a child with an ND, especially during the first 10 years, may have exerted unique demands on the family that reduced their odds of moving out of hardship.

Taken together, the findings are consistent with the social causation framework, which suggests that social conditions before birth are what explain future economic trajectories. This contrasts with the health selection framework, which posits that the poor health condition is the cause of poverty. Similar to Shahtahmasebi *et al*. (2010), our findings lend support for the concern that previous cross‐sectional studies that documented large differences in poverty between ND families and non‐ND families (see, e.g. Montes & Halterman 2008) may have overestimated the influence of ND on family economic well‐being.

Disentangling the relation between ND status and family economic well‐being is challenging primarily for selection reasons. Several studies have established that families that have a child with an ND are more likely to be poor than families raising children who do not have an ND (Emerson & Hatton 2009). A strength of this study is that the rich intergenerational panel design of the PSID allowed us to estimate the economic impacts of having a child with ND in ways that other studies have not. We examined the economic, social and health circumstances at birth and 5 years prior to the birth of the child and analysed prospective poverty and economic hardship trajectories.

Some limitations of the study should be noted. First, the measure of ND includes some caveats. Disability status was based on parents' reports and did not include all forms of ND such as attention‐deficit hyperactivity disorder. As a result, observed differences may be conservatively biased. Further, we were not able to observe the severity of the functional limitations associated with the ND condition or when the ND began. Research has demonstrated that the severity of a disability is strongly associated with experiencing labour market problems (Burton & Phipps 2009). We are not aware of any research showing different trajectories across types of ND or severity and note this is an important line of future research using longitudinal designs. Second, we assumed that those persons most knowledgeable about the child – primary and secondary caregivers – hold financial responsibility for children. It is possible that in a very small amount of cases, children may have resided in complex families where the caregivers interviewed in the PSID were not financially responsible for the child. Unfortunately, there is no way in the PSID to link children to the specific adults who are financially responsible for them.

Third, macroeconomic conditions such as economic growth or recession might have affected poverty and economic hardship. However, we did control for childbirth year, which controlled for some period effect. Fourth, we did not control for state‐level social assistance and other social welfare policies that were designed to offset and mitigate poverty risk. American social welfare assistance is increasingly devolved from federal responsibility to states, and we would expect these to have some impact on poverty risk (Bruch *et al*. 2016). Further, although we investigated baseline socio‐economic variables, future analysis should examine changes in these variables over time. Lastly, the study may suffer an omitted variable bias where factors that affect both economic circumstances and ND status were unmeasured.

In summary, these findings provide new insights into child ND and its links to family economic well‐being. Findings contribute descriptive evidence on the economic circumstances of families with a child with ND. In comparing families raising a child with ND to non‐ND families, we document inequalities that pre‐exist the child's birth and that remain throughout childhood and adolescence. Given the inequality in higher poverty risk, future research should seek to understand other economic factors and policy implications. For example, indicators of financial well‐being such as financial assets and net worth/wealth (Parish *et al*. 2010) could be studied in a prospective longitudinal design to better understand broader dimensions of well‐being. Further, it is crucial to understand the role of social policies designed to offset inadequate market earnings. To what extent do income maintenance, tax credits and social transfers offset the unique poverty risk of families raising a child with an ND? These policy questions are of primary importance given the obligations under the United Nations (1975) Declaration on the Rights of Disabled Persons and the Americans with Disabilities Act.

## Source of Funding

The collection of PSID data used in this study was partly supported by the National Institutes of Health under grant number R01 HD069609 and the National Science Foundation under award number 1157698. This research was supported by a grant from the Kids Brain Health Network of Canada. G.G. was supported by funding from the Canadian Institutes of Health Research.

## Conflict of Interest

No conflicts of interest have been declared

## Supporting information

Figure S1. Trajectory of probability of family living below the poverty threshold and economic hardship, by age of child and ND status of child extended to 5 years before childbirth, modelled from unconditional latent growth models.
Table S1. Detailed results from pooled logistic regressions supporting Figure 1.
Table S2. Detailed results from latent growth curve models supporting Supporting Information Figure 1.
Table S3. Fit statistics and details of the 1‐ to 6‐class solutions for poverty trajectories
Table S4. Output from the selected latent class growth model for poverty trajectories, 5‐class solution (n = 3317)
Table S5. Fit statistics and details of the 1‐ to 6‐class solutions for economic hardship trajectories
Table S6. Output from the selected latent class growth model for economic hardship trajectories, 5‐class solution (n = 3317)
Table S7. Five trajectories of poverty and the associated family and child characteristics.
Table S8. Five trajectories of economic hardship and the associated family and child characteristics.
