# Supplementary material for: Trajectories of poverty and economic hardship among American families supporting a child with a neurodisability
Source: J Intellect Disabil Res. 2019 Jul 12;63(10):1273–84. doi: 10.1111/jir.12666 (PMC6771969; doi:10.1111/jir.12666)
Supplement: Supplementary file 1 — Figure S1. Trajectory of probability of family living below the poverty threshold and economic hardship, by age of child and ND status of child extended to 5 years before childbirth, modelled from unconditional latent growth models. Table S1. Detailed results from pooled logistic regressions supporting Figure 1. Table S2. Detailed results from latent growth curve models supporting Supporting Information Figure 1. Table S3. Fit statistics and details of the 1‐ to 6‐class solutions for poverty trajectories Table S4. Output from the selected latent class growth model for poverty trajectories, 5‐class solution (n = 3317) Table S5. Fit statistics and details of the 1‐ to 6‐class solutions for economic hardship trajectories Table S6. Output from the selected latent class growth model for economic hardship trajectories, 5‐class solution (n = 3317) Table S7. Five trajectories of poverty and the associated family and child characteristics. Table S8. Five trajectories of economic hardship and the associated family and child characteristics. [file JIR-63-1273-s001.docx]

**
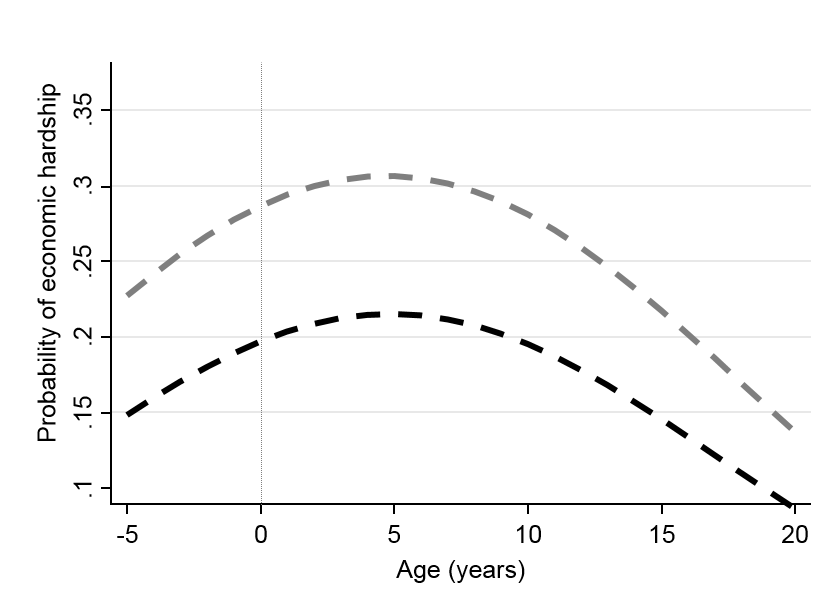
**
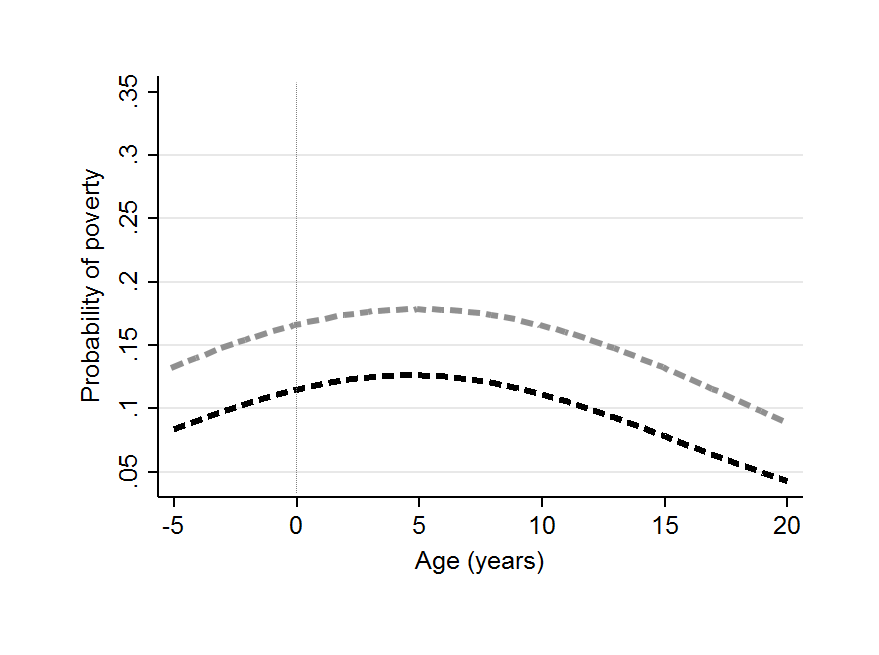
**Supporting Information Figure 1.** Trajectory of probability of family living below the poverty threshold and economic hardship, by age of child and ND status of child extended to 5 years before child birth, modelled from unconditional latent growth models.

a) b)

Legend. a) Trajectory of probability of family living below the poverty threshold; b) Trajectory of probability of family living at or below 150% income to need ratio. Models were weighted using household survey weights. Vertical dashed line indicates year of birth (age 0).

**Supporting Information Table 1.** Detailed results from pooled logistic regressions supporting Figure 1.

|  |  | **Supporting Figure 1a** | | **Supporting Figure 1b** | |
| --- | --- | --- | --- | --- | --- |
|  |  | Probability of family living below the poverty threshold | | Probability of family living living at or below 150% income to need ratio | |
| Variable |  | **Coefficient** | **SE** | **Coefficient** | **SE** |
| Child has a ND |  | 0.170 | 0.203 | 0.434 | 0.155 |
| Child’s age | -5 (ref) | **--** | **--** | **--** | **--** |
|  | -4 | -0.069 | 0.133 | -0.078 | 0.106 |
|  | -3 | -0.055 | 0.131 | 0.037 | 0.105 |
|  | -2 | -0.044 | 0.131 | -0.036 | 0.104 |
|  | -1 | -0.012 | 0.134 | -0.007 | 0.105 |
|  | 0 | 0.224 | 0.127 | 0.216 | 0.101 |
|  | 1 | 0.371 | 0.125 | 0.364 | 0.099 |
|  | 2 | 0.335 | 0.126 | 0.385 | 0.100 |
|  | 3 | 0.441 | 0.127 | 0.380 | 0.101 |
|  | 4 | 0.404 | 0.130 | 0.319 | 0.104 |
|  | 5 | 0.299 | 0.132 | 0.302 | 0.104 |
|  | 6 | 0.456 | 0.130 | 0.429 | 0.103 |
|  | 7 | 0.213 | 0.136 | 0.247 | 0.107 |
|  | 8 | 0.348 | 0.131 | 0.301 | 0.106 |
|  | 9 | 0.173 | 0.142 | 0.218 | 0.112 |
|  | 10 | 0.066 | 0.134 | 0.118 | 0.110 |
|  | 11 | 0.071 | 0.148 | 0.135 | 0.118 |
|  | 12 | 0.076 | 0.144 | 0.059 | 0.114 |
|  | 13 | -0.211 | 0.164 | -0.013 | 0.130 |
|  | 14 | -0.420 | 0.156 | -0.128 | 0.121 |
|  | 15 | -0.204 | 0.165 | -0.094 | 0.133 |
|  | 16 | -0.266 | 0.162 | -0.200 | 0.122 |
|  | 17 | -0.332 | 0.177 | -0.185 | 0.138 |
|  | 18 | -0.602 | 0.174 | -0.586 | 0.137 |
|  | 19 | -0.815 | 0.222 | -0.719 | 0.179 |
|  | 20 | -0.448 | 0.196 | -0.603 | 0.152 |
| Age*ND | -5 (ref) | **--** | **--** | **--** | **--** |
|  | -4 | 0.526 | 0.276 | 0.162 | 0.221 |
|  | -3 | 0.552 | 0.271 | 0.168 | 0.217 |
|  | -2 | 0.432 | 0.273 | 0.103 | 0.216 |
|  | -1 | 0.218 | 0.282 | 0.027 | 0.217 |
|  | 0 | 0.368 | 0.267 | 0.139 | 0.211 |
|  | 1 | 0.043 | 0.275 | 0.033 | 0.210 |
|  | 2 | 0.149 | 0.274 | -0.079 | 0.214 |
|  | 3 | 0.036 | 0.270 | -0.142 | 0.215 |
|  | 4 | 0.016 | 0.279 | -0.087 | 0.220 |
|  | 5 | 0.363 | 0.279 | 0.190 | 0.217 |
|  | 6 | 0.208 | 0.280 | 0.084 | 0.218 |
|  | 7 | 0.317 | 0.290 | 0.050 | 0.226 |
|  | 8 | 0.341 | 0.290 | 0.065 | 0.228 |
|  | 9 | 0.251 | 0.300 | 0.044 | 0.232 |
|  | 10 | 0.543 | 0.293 | 0.2043 | 0.232 |
|  | 11 | 0.188 | 0.303 | -0.087 | 0.240 |
|  | 12 | 0.445 | 0.308 | 0.104 | 0.245 |
|  | 13 | 0.219 | 0.334 | -0.058 | 0.268 |
|  | 14 | 0.676 | 0.322 | 0.103 | 0.258 |
|  | 15 | 0.186 | 0.333 | 0.006 | 0.268 |
|  | 16 | 0.708 | 0.323 | 0.296 | 0.252 |
|  | 17 | 0.175 | 0.348 | -0.348 | 0.275 |
|  | 18 | 0.818 | 0.328 | 0.431 | 0.267 |
|  | 19 | 0.280 | 0.421 | -0.164 | 0.340 |
|  | 20 | 0.288 | 0.421 | -0.116 | 0.336 |
| Constant |  | -2.26146 | 0.092531 | -1.61957 | 0.073 |

**Supporting Information Table 2.** Detailed results from latent growth curve models supporting Supporting Information Figure 1.

| **Supporting Figure 1a** | | | **Supporting Figure 1b** | | |
| --- | --- | --- | --- | --- | --- |
| Probability of family living below the poverty threshold | | | Probability of family living living at or below 150% income to need ratio | | |
|  |  | **Est. (SE)** |  |  | **Est. (SE)** |
| **Family without a child with a ND** |  |  | **Family without a child with a ND** |  |  |
|  | Intercept | -2.04(0.06) |  | Intercept | -1.40(0.05) |
|  | Linear | 0.05(0.01) |  | Linear | 0.05(0.01) |
|  | Quadratic | 0.00(0.00) |  | Quadratic | 0.00(0.00) |
| **Family with a child with a ND** |  |  | **Family with a child with a ND** |  |  |
|  | Intercept | -1.62(0.10) |  | Intercept | -0.91(0.09) |
|  | Linear | 0.04(0.02) |  | Linear | 0.04(0.01) |
|  | Quadratic | 0.00(0.00) |  | Quadratic | 0.00(0.00) |

**Supporting Information Table 3.** Fit statistics and details of the 1- to 6-class solutions for poverty trajectories

| Number of classes | BIC | Polynomial order of coefficients | Group membership percentage (%) of each class |
| --- | --- | --- | --- |
| 1 | 18809.77 | 2 | 100 |
| 2 | 13946.86 | 2 2 | 69.8, 30.2 |
| 3 | 13454.97 | 2 2 2 | 62.5, 34.1, 13.4 |
| 4 | 13259.29 | 2 2 2 2 | 9.6, 64.2, 16.4, 9.9 |
| 5 (selected) | **13019.53** | **2 2 2 2 2** | **14.1, 57.4, 13.5, 6.5, 8.6** |
| 6 | 12849.75 | 2 2 2 2 2 2 | 8.8, 59.1, 9.5, 7.9, 6.0, 8.7 |
| *Note.* The 5-class model was selected and discussed in the manuscript (see Figure 2a). | | | |

**Supporting Information Table 4.** Output from the selected latent class growth model for poverty trajectories, 5-class solution (n=3317)

| Group trajectory | Parameter | Estimates | Standard error | P-value |
| --- | --- | --- | --- | --- |
| 1  Fast exit out of poverty | Intercept | -1.419 | 0.636 | 0.026 |
|  | Linear | -0.116 | 0.142 | 0.411 |
|  | Quadratic | 0.001 | 0.013 | 0.965 |
|  |  |  |  |  |
| 2  Persistent non-poverty | Intercept | -4.540 | 0.292 | 0.000 |
|  | Linear | 0.023 | 0.094 | 0.810 |
|  | Quadratic | -0.001 | 0.006 | 0.892 |
|  |  |  |  |  |
| 3  Transient poverty | Intercept | -1.175 | 0.162 | 0.000 |
|  | Linear | 0.278 | 0.045 | 0.000 |
|  | Quadratic | -0.016 | 0.001 | 0.000 |
|  |  |  |  |  |
| 4  Slow exit out of poverty | Intercept | 1.184 | 0.345 | 0.001 |
|  | Linear | -0.218 | 0.048 | 0.000 |
|  | Quadratic | 0.001 | 0.004 | 0.854 |
|  |  |  |  |  |
| 5  Persistent poverty | Intercept | 1.363 | 0.176 | 0.000 |
|  | Linear | 0.275 | 0.045 | 0.000 |
|  | Quadratic | -0.019 | 0.003 | 0.000 |
| *Note.* Estimates refer to the 5-class model discussed in text and displayed in Figure 2a. Variance-covariance matrices were estimated. Due to space limitations they are not presented here but available from authors. | | | | |

**Supporting Information Table 5.** Fit statistics and details of the 1- to 6-class solutions for economic hardship trajectories

| Number of classes | BIC | Polynomial order of coefficients | Group membership percentage (%) of each class |
| --- | --- | --- | --- |
| 1 | 26435.54 | 2 | 100 |
| 2 | 19343.72 | 2 2 | 58.2, 41.8 |
| 3 | 18187.80 | 2 2 2 | 49.4, 30.3, 20.3 |
| 4 | 17702.93 | 2 2 2 2 | 49.5, 14.4, 17.6, 18.5 |
| 5 (selected) | **17472.62** | **2 2 2 2 2** | **7.5, 49.0, 14.3, 10.4, 18.7** |
| 6 | 17370.98 | 2 2 2 2 2 2 | 10.3, 45.9, 10.3, 7.5, 9.8, 16.1 |
| *Note.* The 5-class model was selected and discussed in the manuscript (see Figure 2b). | | | |

**Supporting Information Table 6.** Output from the selected latent class growth model for economic hardship trajectories, 5-class solution (n=3,317)

| Group trajectory | Parameter | Estimates | Standard error | P-value |
| --- | --- | --- | --- | --- |
| 1  Short transient hardship | Intercept | -0.042 | 0.235 | 0.858 |
|  | Linear | 0.428 | 0.056 | 0.000 |
|  | Quadratic | -0.064 | 0.010 | 0.000 |
|  |  |  |  |  |
| 2  Persistent non-hardship | Intercept | -3.652 | 0.091 | 0.000 |
|  | Linear | -0.056 | 0.022 | 0.011 |
|  | Quadratic | 0.002 | 0.001 | 0.131 |
|  |  |  |  |  |
| 3  Long transient hardship | Intercept | -1.171 | 0.164 | 0.000 |
|  | Linear | 0.294 | 0.031 | 0.000 |
|  | Quadratic | -0.014 | 0.001 | 0.000 |
|  |  |  |  |  |
| 4  Slow exit out of hardship | Intercept | 0.019 | 0.194 | 0.921 |
|  | Linear | -0.252 | 0.029 | 0.000 |
|  | Quadratic | 0.005 | 0.002 | 0.024 |
|  |  |  |  |  |
| 5  Persistent hardship | Intercept | 1.912 | 0.142 | 0.000 |
|  | Linear | 0.115 | 0.027 | 0.000 |
|  | Quadratic | -0.012 | 0.001 | 0.000 |
| *Note.* Estimates refer to the 5-class model discussed in text and displayed in Figure 2a. Variance-covariance matrices were estimated. Due to space limitations they are not presented here but available from authors. | | | | |

**Supporting Information Table 7.** Five trajectories of poverty and the associated family and child characteristics*.*

|  | Trajectory 1 | Trajectory 2 | Trajectory 3 | Trajectory 4 | Trajectory 5 |
| --- | --- | --- | --- | --- | --- |
|  | Persistent non-poverty | Fast exit out of poverty | Transient poverty | Slow exit out of poverty | Persistent poverty |
| % of the sample | 57.4% | 14.1% | 13.5% | 6.5% | 8.6% |
|  | % (n) | % (n) | % (n) | % (n) | % (n) |
| Child has a ND | 17.5 (405) | 24.2 (81) | 26.2 (101) | 32.6 (39) | 28.8 (45) |
| Child gender |  |  |  |  |  |
| Male | 48.9 (1079) | 56 (181) | 49.2 (173) | 55.4 (65) | 47.4 (69) |
| Female | 51.1 (1126) | 44 (143) | 50.8 (179) | 44.6 (52) | 52.6 (77) |
| Age of PCG, mean (SD) | 28.9 (6) | 26.3 (6.2) | 25.7 (6.7) | 25.9 (6.6) | 26.8 (8.7) |
| Race of PCG |  |  |  |  |  |
| White | 78.8 (1827) | 62.9 (210) | 47.7 (184) | 34.6 (42) | 21.9 (34) |
| Not white | 21.2 (492) | 37.1 (124) | 52.3 (202) | 65.4 (78) | 78.1 (123) |
| Years of education of PCG, mean (SD) | 14.2 (2.4) | 13.4 (2.1) | 12.7 (2.8) | 12.9 (2.1) | 11.3 (2.8) |
| Marital status of PCG |  |  |  |  |  |
| Married/partnered | 89.7 (1790) | 61.4 (180) | 54.5 (153) | 24.5 (27) | 30.2 (36) |
| Not married/partnered | 10.3 (206) | 38.6 (113) | 45.5 (128) | 75.5 (84) | 69.8 (83) |
| Working status of PCG |  |  |  |  |  |
| Not Working | 32.4 (575) | 52.2 (133) | 55.3 (129) | 71.2 (67) | 71.6 (75) |
| Working | 67.6 (1201) | 47.8 (122) | 44.7 (104) | 28.8 (27) | 28.4 (30) |
| Self-rated health of PCG |  |  |  |  |  |
| Excellent | 39.5 (788) | 21.9 (65) | 15.4 (43) | 21.5 (24) | 15 (18) |
| Very good | 38.9 (776) | 41.3 (123) | 35.7 (100) | 31.5 (35) | 22 (27) |
| Good | 18.1 (360) | 27.4 (82) | 32 (89) | 29.2 (33) | 32.6 (40) |
| Fair | 2.7 (53) | 6 (18) | 14.1 (39) | 15.5 (17) | 25.8 (31) |
| Poor | 0.8 (15) | 3.4 (10) | 2.7 (8) | 2.3 (3) | 4.6 (6) |
| Number of children in household, mean (SD) | 1.5 (1) | 1.8 (1.1) | 1.7 (1.2) | 2.6 (1.5) | 2.4 (1.3) |

*Note.* The five trajectories are based on Model 5, Supporting Information Table 1. Characteristics of the child and the primary caregiver (PCG) based on the year prior to childbirth. Analyses do not include the functional forms of the trajectories.

**Supporting Information Table 8.** Five trajectories of economic hardship and the associated family and child characteristics*.*

|  | Trajectory 1 | Trajectory 2 | Trajectory 3 | Trajectory 4 | Trajectory 5 |
| --- | --- | --- | --- | --- | --- |
|  | Persistent non-hardship | Short transient hardship | Long transient hardship | Slow exit out of hardship | Persistent hardship |
| % of the sample | 49.0% | 7.5% | 14.3% | 10.4% | 18.7% |
|  | % (n) | % (n) | % (n) | % (n) | % (n) |
| Child has a ND | 16.7 (331) | 23.4 (55) | 25.1 (104) | 27 (67) | 26.4 (115) |
| Child gender |  |  |  |  |  |
| Male | 49.9 (944) | 49.7 (111) | 51.3 (199) | 50.8 (118) | 48.1 (195) |
| Female | 50.1 (949) | 50.3 (112) | 48.7 (189) | 49.2 (115) | 51.9 (211) |
| Age of PCG, mean (SD) | 29.4 (6) | 26.5 (5.4) | 26.8 (6.6) | 25.1 (5.7) | 25.9 (7.3) |
| Race of PCG |  |  |  |  |  |
| White | 82.3 (1632) | 69.6 (163) | 55.9 (232) | 62.8 (156) | 26.5 (116) |
| Not white | 17.7 (352) | 30.4 (71) | 44.1 (183) | 37.2 (92) | 73.5 (321) |
| Years of education of PCG, mean (SD) | 14.6 (2.1) | 13.3 (2.4) | 12.7 (2.3) | 13.3 (1.7) | 11.6 (3.1) |
| Marital status of PCG |  |  |  |  |  |
| Married/partnered | 90.9 (1599) | 66.5 (128) | 71.7 (228) | 54.3 (132) | 34.4 (99) |
| Not married/partnered | 9.1 (159) | 33.5 (65) | 28.3 (90) | 45.7 (112) | 65.6 (189) |
| Working status of PCG |  |  |  |  |  |
| Not Working | 30.5 (480) | 53.9 (91) | 43.8 (113) | 56 (118) | 70.5 (177) |
| Working | 69.5 (1094) | 46.1 (78) | 56.2 (145) | 44 (93) | 29.5 (74) |
| Self-rated health of PCG |  |  |  |  |  |
| Excellent | 41.6 (732) | 23.4 (45) | 17.4 (55) | 26.3 (64) | 14.6 (42) |
| Very good | 38 (668) | 45.8 (88) | 42.4 (134) | 38.8 (94) | 26 (76) |
| Good | 17.4 (306) | 26.9 (51) | 30.5 (97) | 23 (56) | 32.3 (94) |
| Fair | 2.5 (44) | 3 (6) | 8.4 (27) | 7.7 (19) | 22.1 (64) |
| Poor | 0.6 (11) | 0.9 (2) | 1.3 (4) | 4.1 (10) | 5 (14) |
| Number of children in household, mean (SD) | 1.5 (1) | 1.8 (1) | 1.5 (1.1) | 2 (1.2) | 2.4 (1.4) |

*Note.* Characteristics based on the five-class model (see Supporting Information Table 2). Characteristics of the child and the primary caregiver (PCG) based on the year prior to childbirth. Analyses do not include the functional forms of the trajectories.
